# “It's All Sort of Cool and Interesting…but What Do I Do With It?” A Qualitative Study of Stroke Survivors' Perceptions of Surface Electromyography

**DOI:** 10.3389/fneur.2020.01037

**Published:** 2020-09-17

**Authors:** Heather A. Feldner, Christina Papazian, Keshia Peters, Katherine M. Steele

**Affiliations:** ^1^Department of Rehabilitation Medicine, University of Washington, Seattle, WA, United States; ^2^Department of Mechanical Engineering, University of Washington, Seattle, WA, United States

**Keywords:** surface electromyography, stroke, qualitative research, perceptions of technology, rehabilitation

## Abstract

**Background:** Stroke is one of the most common neurologic injuries worldwide. Over decades, evidence-based neurorehabilitation research and advancements in wireless, wearable sensor design have supported the deployment of technologies to facilitate recovery after stroke. Surface electromyography (sEMG) is one such technology, however, clinical application remains limited. To understand this translational practice gap and improve clinical uptake, it is essential to include stakeholder voices in an analysis of neurorehabilitation practice, the acceptability of current sEMG technologies, and facilitators and barriers to sEMG use in the clinic and the community. The purpose of this study was to foreground the perspectives of stroke survivors to gain a better understanding of their experiences in neurorehabilitation, the technologies they have used during their recovery, and their opinions of lab-designed and commercially-available sEMG systems.

**Methods:** A qualitative, phenomenological study was completed. In-depth, semi-structured interviews were conducted with eight stroke survivors (age range 49–78 years, 6 months to 12 years post-stroke) and two caregivers from a large metropolitan region. A demonstration of four sEMG systems was provided to gather perceptions of sensor design, features and function, and user interface. Interviews were audio-recorded, transcribed verbatim, and coded for analysis using constant comparison until data saturation was reached.

**Results:** Three themes emerged from the data: (1) “*Surface EMG has potential….but…*” highlights the recognition of sEMG as a valuable tool but reveals a lack of understanding and need for clear meaning from the data; (2) “*Tracking incremental progress over days or years is important*” highlights the persistence of hope and potential benefit of sEMG in detecting small changes that may inform neurorehabilitation practice and policy; and (3) “*Neurorehabilitation technology is cumbersome*” highlights the tension between optimizing therapy time and trying new technologies, managing cost, logistics and set-up, and desired technology features.

**Conclusion:** Further translation of sEMG technology for neurorehabilitation holds promise for stroke survivors, but sEMG system design and user interface needs refinement. The process of using sEMG technology and products must be simple and provide meaningful insight to recovery. Including stroke survivors directly in translational efforts is essential to improve uptake in clinical environments.

## Introduction

Over the past decades, there has been a prolific amount of research and development of technology to enhance both the understanding of neurologic injuries and the application of evidence-based neurorehabilitation interventions. Surface electromyography (sEMG) is one such technology that has undergone rapid advancement in development, but has yet to reach its full translational potential to help drive neurorehabilitation and maximize recovery. Understanding this translational gap must consider multiple factors across a complex landscape of healthcare provision, especially given the public/private healthcare model in the United States. Successful deployment of sEMG in clinical environments relies on an interaction of system design, funding, translational research findings, clinician training, and user acceptance, among many other factors. While user acceptance of neurorehabilitation technology is just a small piece of a much larger puzzle, it is an essential one, and a more explicit understanding of the perceptions and experiences of individuals with neurologic injury, such as stroke, is warranted to better understand the barriers, facilitators, and untapped potential of sEMG technology in clinical neurorehabilitation,

Stroke is one of the most common neurologic injuries worldwide (1, 2). Recent global statistics estimate nearly 14 million new instances of stroke annually; stroke related healthcare costs in the US alone have topped $750 billion annually and are projected to increase as a result of the aging population (3, 4). Further, the psychosocial and functional impacts of stroke are also significant, leading to stress, isolation, and potential comorbid health conditions (5, 6). While neurorehabilitation is a central feature of recovery for individuals with stroke, outcomes can be disparate and long-term impairment is common, further influenced by the extent to which stroke survivors have the geographic, financial, healthcare, and socio-emotional resources to maximize recovery following their injury (1). It is because of this significant impact of stroke at both individual and institutional levels that the field of neurorehabilitation must engage in a deeper exploration of the translation of advanced healthcare technologies into clinical settings to enhance our knowledge and provision of care during recovery from neurologic injuries.

Surface EMG today is used in research and clinical environments across a wide variety of physiological and engineering applications relating to rehabilitation, sport performance, occupational performance, and beyond (7). More specific to neurorehabilitation, foundational literature in the mid-twentieth century described sEMG as a useful tool to characterize neuromuscular patterns, demonstrated the relative contribution of different muscles in functional movement, and in some cases, assisted in prognosis of recovery following neurologic injury (8, 9). Across many subsequent decades, researchers have used sEMG to examine factors in participants with and without neurologic impairments such as interlimb coordination, muscle activation and co-activation patterns, response to biofeedback, and most recently, as a tool to determine treatment appropriateness and costs in stroke survivors with gait impairments (7, 10–14). Despite these advances, a significant body of literature supporting the use of sEMG, and the establishment of expert guidelines for sEMG implementation through SENIAM (Surface EMG Non-Invasive Assessment of Muscles), a lack of clinical translation of sEMG technology has also been recognized by researchers (7, 15–18).

One potential reason for the slow clinical uptake of sEMG and related neurorehabilitation technologies may be the paucity of perspectives in research from clinicians as providers of sEMG assessment or intervention, and individuals with neurologic conditions and their caregivers as recipients of sEMG assessment or intervention. Considering sEMG alongside other neurorehabilitation technologies more broadly, the literature is lacking a clear picture of how and how often these technologies are used in clinics across the US, and how technology users and their caregivers respond to the design, logistics of use, and output of the devices. However, user and caregiver perspectives are a key untapped resource in the design and implementation of rehabilitation technologies such as sEMG, and have the potential to richly contextualize the barriers and facilitators that affect technology acceptance and use. For example, within the broader realm of neurorehabilitation technology, Alt Murphy et al. (19) recently published a qualitative analysis of participant responses to a novel wearable sensor garment to monitor physiologic and movement parameters for individuals with stroke, Parkinson's Disease, or Epilepsy. The authors reported that responses to the upper body garment was acceptable, but participants noted challenges with fit and comfort and felt uncertain about consistent monitoring and privacy (19). Another study noted similar comfort issues with wearable sensors, but highlighted that despite the discomfort, participants preferred the automated data tracking features of the sensors compared to more time-intensive activities such as completing activity or symptom diaries (20).

Additional qualitative work with stroke survivors and clinicians has also explored perspectives and experiences of the rehabilitation process itself, as well as technologies such as virtual reality, gaming, robotic exoskeletons, or other wearable devices, but little work has focused specifically on sEMG (21–29). One study included gaming as part of a structured, enriched rehabilitation environment, which garnered positive responses from participants who noted increased motivation to move as well as friendly competition between other participants on the unit (29). Perceptions of virtual reality systems varied, with one study reporting low rates of side effects but high rates of perceived exertion by stroke survivors (21), and another describing how users felt enjoyment and motivation using a novel technology they would not otherwise have had access to, but felt that the experiences with virtual reality did not translate into improved functional carryover (23). Many studies have examined robotic applications for stroke rehabilitation, but very few have included survivor perspectives. Those that have describe user priorities of cost, better movement quality, endurance, practicality, and appropriate training and support, but also highlight technology acceptance issues as a potential barrier for clinical or home use (30–33). One set of studies investigated the preliminary use of sEMG as a control mechanism for a gaming system in chronic stroke survivors, finding significant pre and post intervention sEMG changes, and qualitative outcomes which indicated most participants would recommend neurogaming to others for enjoyment, despite a lack of reported functional carryover (26, 34). Our recent work has explored rehabilitation clinicians' perspectives of the use of sEMG in practice with individuals with neurologic conditions, who noted the potential benefits of objective recovery tracking, muscle training, and patient motivation, but also acknowledged barriers to sEMG use such as time, training, and access to funds and technical support for sEMG equipment (35).

The literature notes that the introduction of novel healthcare technologies into existing clinical practices can be challenging, as the process often disrupts engrained care routines (36). Resistance to new technology integration, as well as distinct ways of evaluating the utility of technology from professional and lay perspectives are common (37). This has consequences for both healthcare providers as well as patients. For example, healthcare providers have noted translational difficulties, including challenges with clearly communicating results to patients and using technology outputs to meaningfully guide treatment decisions. Patients have expressed uncertainty about the purpose of technology as a part of their care, and a failure to receive meaningful results from their providers (37). Applied to rehabilitation, it is reasonable to expect that there may be similar challenges when considering the implementation of sEMG technology, especially considering the introduction of a high-tech, objective, instrumented assessment tool juxtaposed with clinical standards that typically involve low-tech, subjective, scaled tools such as manual muscle testing or dynamometry. Experiences such as these underscore that clinician training, communication about technology intent, impact, and translational capacity to assist in healthcare decision-making are important factors to consider in improving uptake of technology in clinical settings.

The purpose of this early-stage study was to foreground the perspectives of stroke survivors and gain a better understanding of their experiences in neurorehabilitation, the technologies they have used during their recovery, and their introductory perceptions of one lab-designed prototype and three commercially available sEMG systems. Centering these perspectives is critical to understanding the barriers and untapped potential of sEMG and other neurorehabilitation technologies that may support the recovery of individuals with neurologic injuries. This qualitative work complements and builds upon past milestones in sEMG research across rehabilitation and engineering fields. It offers a preliminary look at baseline user perspectives to inform more robust research in the future, and provides a unique opportunity to leverage user-centered perspectives to support potential innovations in sEMG design, implementation, and outcomes.

## Methods

All procedures in this study were approved by the authors' institutional review board, and written consent was obtained by all participants prior to initiation of study procedures. Participant names are pseudonyms to protect privacy and confidentiality. This qualitative study was conducted using a phenomenological approach, which is ideal for understanding the lived experiences of a group of participants with similar characteristics (38). Ultimately, the goal of phenomenological research is to describe and interpret a given phenomenon through the lens of individuals with first-hand knowledge of the event or experience, in this case, the experience of having and living with stroke and experiencing neurorehabilitation (39, 40). While this lived experience may or may not have included technology use during recovery, it provided a shared foundation from which to obtain informed perceptions of sEMG as a potential part of this experience.

### Research Team Background

This study was conducted by a multidisciplinary research team, including a physical therapist with qualitative research expertise, mechanical engineer with wearable sensor expertise, and two research scientists. All members of the team had extensive training and experience in the clinical application of sEMG systems to track muscle activity changes in stroke survivors. In addition to this qualitative work, the research team was concurrently collecting sEMG data from stroke survivors in acute care and community-based settings. Therefore, the researchers were well-positioned to engage with and provide baseline information to the participants about sEMG technology.

### Study Procedures

**Figure 1:**
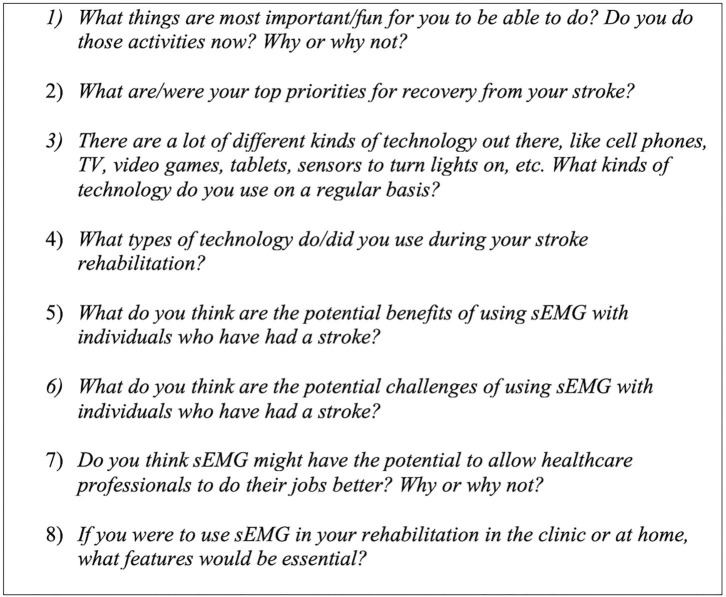
Sample Semi-Structured Interview Questions: These questions were among those asked of each participant during the semi-structured interview. Responses were audio-recorded and transcribed verbatim.

Semi-structured interviews were conducted to obtain primary source thoughts, opinions, and interpretations from the participants, using an interview guide that was developed by the authors, edited for content until consensus was reached, and piloted with a volunteer to ensure clarity of question content and order. Figure 1 contains a list of sample questions from the interview guide.

**Figure 2:**
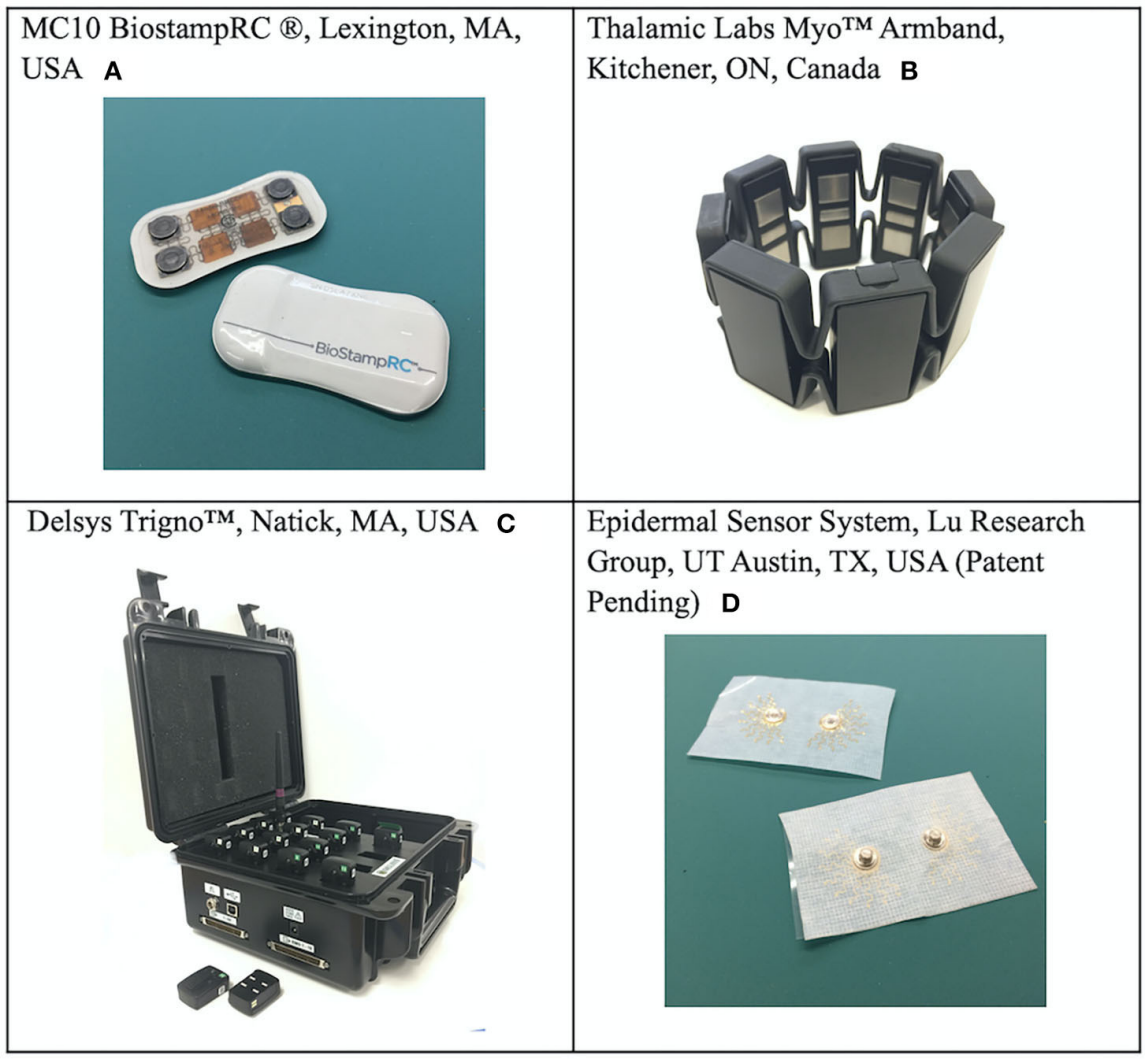
Sample Commercial and Lab-Based sEMG Sensors: A brief demonstration and feature discussion for these four sEMG systems was conducted during each participant interview. Systems were available for physical inspection, but not applied to the participants. **(A)** MC10 Biostamp® **(B)** Thalamic Labs Myo™ Armband; **(C)** Delsys Trigno™; and **(D)** Epidermal Sensor System (patent pending).

During each interview, a brief demonstration of four, research lab-owned sEMG systems was conducted. This included one lab-designed sEMG sensor prototype as well as three commercially available sEMG systems, chosen to represent a broad range of system design, aesthetic, and capabilities: (A) MC10 BiostampRC® (Lexington, MA, USA); (B) Thalamic Labs Myo™ Armband (Kitchener, Ontario, CAN); (C) Delsys Trigno™ (Natick, MA, USA); and (D) Epidermal Sensor System (Austin, TX, USA, patent pending), a lab-designed prototype with sensor filament embedded in medical tape. See Figure 2 for images of each sEMG system. Participants were able to examine each system and were briefed on features including functionality and purpose, battery life, skin preparation needs, anatomical placement, user interface, and cost. Participants were also oriented to print versions of sample signal outputs from each system, since time constraints prohibited real-time system use. To minimize the potential for biased responses, the research team refrained from endorsing any given system and only provided pre-scripted, general purpose information about each system and the clinical applications of sEMG to assist the participants in offering informed perceptions. Participants were able to ask clarifying questions about the purpose and features of the systems, and self-assessed their understanding of the information presented prior to continuing the interview. Feedback about the features and perceived utility of each sEMG system was then solicited from each participant.

Interviews were conducted at a location of the participant's choosing, with half the interviews taking place at the participant's home, and half taking place in university settings such as an office or research lab. All interviews were audio-recorded, transcribed verbatim, and de-identified.

### Participants

Participants were recruited via convenience sampling through stroke clinics and rehabilitation professional contacts across a large metropolitan area. To be included in the study, participants must have been over the age of 18 years, have the cognitive capability to consent for themselves, have had any type of stroke in the past or be the spouse or caregiver of the stroke survivor, and demonstrate proficiency communicating in English. Potential participants were excluded from the study if they were under the age of 18 years, not able to cognitively consent for themselves, or communicate proficiently in English. Stroke survivors with aphasia or limited communication capabilities were included in the study along with their spouses/caregivers. Ten participants across the Seattle metropolitan region completed the study, including eight stroke survivors and two spouses/caregivers. Of the stroke survivors, four were male and four were female, ranging in age from 49 to 78 years old (mean = 65 years), and ranging from 6 months to 12 years post-stroke (mean = 4.75 years). One male and one female spouse/caregiver participated in the interviews with their respective partners. Two participants had mild to moderate expressive aphasia, one participated in the interviews independently, and another participated to the greatest extent possible with the assistance of his spouse. Most participants (*n* = 8) had post-secondary vocational training or college degrees, with employment backgrounds including business and finance administration, teaching, musician, and aerospace engineering. One participant had previously worked in a research and development capacity with activity monitoring technology for nearly 20 years, and another had participated in a prior research study using sEMG for serious gaming. The remaining participants did not have prior experience with sEMG, though many had used or trialed related neuromuscular rehabilitation technologies such as electrical stimulation or biofeedback, as well as ubiquitous lay technologies such as commercial fitness or activity trackers, following their stroke.

### Data Analysis

De-identified transcripts were analyzed inductively for their responsiveness to the research purpose, and coded using constant comparison until data saturation was reached and themes grounded in the participants' perceptions and experiences emerged (38, 39). The authors engaged first in open coding, followed by further, independent content analysis and focused coding and discussion among the team to consolidate focused codes into themes. All themes were created with >95% agreement, and any differences in interpretation were resolved by discussion until consensus was achieved. Interview participants engaged in member checking, by which they were provided an opportunity to review a summary of the findings and ask questions so the research team could confirm accuracy and avoid misinterpretation of the results (38). A “thick description” of participant perceptions and experiences, supported directly by verbatim quotations, is presented to allow reader-driven determination of data credibility (39). Table 1 shows a detailed example of the structured coding process.

**Table 1:** Qualitative coding structure.

**Quote**	**Open Coding**	**Focused Coding**	**Theme**
“If you can see things, that would be helpful in acute rehab, cause there is time there. You're just in bed a lot of the time, and that's the time to work, you know? I think seeing linkages between each part of this, like, if the home healthcare people use the same data that they use [in acute rehab], they can come in and say, ‘Well, you were working on this particular muscle so this time let's give more attention there and then do a check and see how these others are doing’. And then you kind of feel that progression with your [program].”	Seeing is believing, a lot of downtime, downtime as worktime, information for therapists, data to drive rehabilitation, progression of rehabilitation	Visualizing objective data, downtime as worktime, technology can drive rehabilitation	sEMG has potential…but
“Remember they said if you don't have any recoverability within the first 3 months, if you don't have it by then…kiss it goodbye. So, I'm fighting. I am making little improvements, not big ones, but little ones. Only because I keep on fighting. You don't see that years out like I am.”	Return to function, timeline on return, seeing progress, little improvements, motivation and persistence over years	Seeing actual change, motivation and persistence to keep working	Tracking incremental progress over days and years is important
“E: And [e stim], it's something he can't do by himself. So, I have to be available to do it with him, and then, you know, it's just very touchy so you have to replace [the pads] a lot during the thing, so it's not just like we can slap it on and let it go. We're both kind of there the whole time, so it takes a lot longer than it should, I think. D: What…have a turn on and be working E: And stay on. Exactly (laughing). It's not even the length of time, it's just having to fix it all the time. Makes us both crazy!”	Can't set up by yourself, need for caregiver presence, equipment is finicky, cost/use of replacement supplies, willing to put in the time, clinical effectiveness	Technology set-up and logistical challenges, time vs. effort	Neurorehabilitation technologies are cumbersome

*This table demonstrates the iterative process of qualitative coding. Beginning with a quote on the left, each quote is annotated with open codes on first reading, which are subsequently streamlined into focused codes on later readings, and finally merged into overarching themes that describe how the focused codes relate to one another*.

## Results

Three major themes emerged from the data: (1) “*Surface EMG has potential.but…*”; (2) “*Tracking incremental progress over days or years is important*”; and (3) “*Neurorehabilitation technology is cumbersome*”. These themes inform an overarching construct that sEMG could be valuable for stroke survivors, but the process and products must be simple and meaningful to their recovery in order to achieve greater uptake in both clinical and community settings during rehabilitation. Within these themes, the participants identified several key features of existing sEMG systems that were appealing, as well as features that presented barriers to use. Participants also offered innovative ideas and solutions for future iterations of sEMG technology and key insights for improved technology translation in healthcare.

### Theme 1: “Surface EMG Has Potential…but…”

The first theme highlights perspectives that sEMG could offer motivation, reinforcement, and provide more precise data during recovery, but this data is only valuable if its output is both meaningful for stroke survivors and clinically useful for rehabilitation professionals. Participants also identified that timing of sEMG application is a key consideration. In general, participants felt that having sEMG data would help clinicians in care planning and decision-making, however, there was some disagreement among participants as to if this was a valuable use of clinicians' time. Ultimately, participants felt excited about the potential of sEMG technology and an integrated approach to rehabilitation that included clinicians, engineers, and patients. However, participants also expressed a simultaneous need for further clarity about the impact of sEMG data on their recovery.

For example, a majority (*n* = 8) participants felt that sEMG could be a useful way to deliver more objective data to support their self-perceived assessment of functional recovery (Table 2, Quotes 1-2). In regard to optimal timing, there were differing opinions as to whether sEMG would be valued in acute rehabilitation prior to return of visible movement. One stroke survivor thought sEMG would be more useful after voluntary movement returned, however, this was an outlying view (Table 2, Quote 3). The remaining participants felt sEMG data could be applied early, to facilitate a more integrated approach to monitoring recovery, and over half of the participants noted that sEMG technology would translate well between clinical and home settings during the recovery process (Table 2, Quotes 4-6).

**Table 2:** Theme 1: 'sEMG has potential.but' Participant Quotes.

**#**	**Quote**	**Description**
1	“If Cherry sees improvement with a device, as opposed to thinking, ‘Gee I thought I walked better today’, You see what I mean. You've got hard data that says, ‘yeah, you did walk better’, okay, reinforcing her mind.” (Archie, spouse).	Visualizing objective data, data-driven recovery, motivation
2	“If you were identifying some task-oriented thing and you thought, ‘Well, I want to be able to reach into that cabinet’, and then each day you could kind of check on how well did you do, I think it would give you data to help you…when you're here, and trying to get there.” (Cherry, 77)	
3	“In my case, when their arm are like this, they're trying to get moving so I don't think those [sensors] would be very good until you get after [moving]. I think after, it would seem to help me see if I can do it or not do it” (Jane, 68).	Seeing change over time
4	“If you can see things, that would be helpful in acute rehab, cause there is time there. You're just in bed a lot of the time, and that's the time to work, you know? I think seeing linkages between each part of this, like, if the home healthcare people use the same data that they use [in acute rehab], they can come in and say, ‘Well, you were working on this particular muscle so this time let's give more attention there and then do a check and see how these others are doing’. And then you kind of feel that progression with your [program], I think.” (Cherry, 77)	Downtime is worktime, monitoring outside of therapy time
5	“And you'd probably take that same [technology] home with you, if you started out in acute rehab with using the equipment.” (Archie, spouse)	Translation from clinic to home
6	“I think it's a great addition and I think once you're done with the bulk of therapy, this might be an easy or a good way to continue things at home.” (Emily, spouse)	
7	“I think it would [help healthcare professionals do their jobs better] and also show progression if something does change. Then they get a database on each patient and can say, ‘here's the normal range, here's where this one is, in this one area what can we do to strengthen’. I think it would increase productivity. It might not decrease the time, but you might be able to do a lot more. It would definitely help the therapists, and also your recovery” (Cherry, 77)	Helpful information for healthcare providers
8	“Too time-consuming…It's not the right type [to use in therapy]” (Daniel, 66)	May interfere with hands-on exercise or functional activities
9	“Yeah, you know, when we go in for therapy with you, when she spends a lot of time playing with the electrodes and the e-stim, and then we leave, it's just like you've used up one Medicare session and you don't feel like you've gotten enough done” (Emily, spouse)	
10	“It's important to involve users as well as the engineers. Because engineers come up with these great ideas, but it's the users who are actually much more precise. With aligning a prosthesis, the prosthetist has been trained and worked to really know how to do that, but if you give the amputee control over alignment, they'll come up much more precise than the prosthetist is, you know?” (Jill, 77)	User empowerment, lived experience, multidisciplinary approach
11	“That would be really cool to have a brainstorming session, so you can go through the logic and opportunities and bringing in IT people, physical therapists, mechanical engineers, and you've got yourself a powerful group to download information. Put a patient in there, you'll have some great, cheap innovations coming.” (Anne, 62)	
12	“I think it's all sort of cool and interesting, but again the question is so what do I do with it? How do I set a goal for myself using it? And, how does it help me improve? How does looking at these graphs or seeing if I can make them look the way they need to look help me improve?” (Duke, 49).	Skepticism about value, interpreting the data
13	“Is it actually worthwhile doing this? Does it work for you? It may work for one person and not another…” (Anne, 62)	
14	“I would need more explanation of what the raw signals mean. What are they actually measuring, and what kind of output do you want, you know, do you want precision or do you want general, cause some people just wonder what's the count for the day, they weren't interested in what your highest was, or they're interested in just one piece of it.” (Jill, 77)	

*This table encapsulates relevant example quotes from the results which supported the development of Theme 1: ‘sEMG has potential…but’*.

A majority of participants (*n* = 8) also highlighted the potential importance of sEMG in providing objective information to their clinical teams, noting that it could fill a gap for medical providers in challenging or ambiguous situations, and assist in making accurate prognostic decisions, providing concrete feedback to survivors and caregivers, or improve rehabilitation productivity (Table 2, Quote 7). One couple, in drawing from their previous experiences with electrical stimulation, had an alternative view, feeling like time spent with set up or monitoring of sEMG could interfere with other therapy activities and reduce time for hands-on functional activities or exercises with therapists (Table 2, Quotes 8-9).

Among over half the participants (*n* = 6), there was recognition of sEMG as a means to invite a multidisciplinary, user-centered technology experience into rehabilitation and recovery. Participants were excited about the prospect of brainstorming sessions to leverage existing technologies and innovate with new ideas, and highlighted the importance of the technology user as a primary stakeholder with lived expertise in regard to these healthcare advances (Table 2, Quotes 10-11). Despite these perceived benefits, reservations among participants remained regarding the interpretation of sEMG outputs and appraising the value of a potential investment in sEMG during recovery (Table 2, Quotes 12-14).

### Theme 2: “Tracking Incremental Progress Over Days or Years Is Important”

The second theme reflects participants' experiences of small changes or progress months and years after stroke, the potential role of technology like sEMG in detecting such change during acute and long-term recovery, and the need for more data to document objective changes that may inform neurorehabilitation policy and practice.

For example, all participants discussed their recovery journey, highlighting processes of both self-discovery as well as harsh realities, noting that they surprised themselves and their medical team with changes long after their stroke. Depending on how recently the stroke occurred, these changes were still emerging (Table 3, Quotes 1-4). Progress was slower than expected, and even with positive experiences in recovery, long adjustment periods and fear were common threads (Table 3, Quote 5). As a part of this incremental recovery, a majority of participants (*n* = 7) noted they would appreciate the precision offered by sEMG and the potential to identify small changes during rehabilitation (Table 3, Quotes 6-7). The participants felt that the capability to monitor muscle activity and see these small changes was a benefit that outweighed the potential for discouragement if minimal or no progress was observed in a particular muscle group (Table 3, Quote 8).

**Table 3:** Theme 2: “Tracking incremental progress over days or years is important.”

**#**	**Quote**	**Description**
1	“With my hand and arm, it's tough, you know, but every once in a while, I'll still see something. I feel like if I can make myself do something like once or twice, you know, its brutally difficult the first couple of times, but once you can do it a few times or a handful of times you can start to get better at doing it consistently.” (Duke, 49)	Progress takes time, small changes are a big deal, motivation to keep fighting
2	“I do exercise every day. Three times a day. I put my stimulator on my hand and it lifts my hand open because I couldn't do anything with my right hand. When I first got home, all I could do was…like that [pulls arm against body]. Now, I can do almost anything, but [my hand] still won't work, but I'm working on it.” (Jane, 68)	
3	“Remember they said if you don't have any recoverability within the first 3 months, if you don't have it by then…kiss it goodbye. So, I'm fighting. I am making little improvements, not big ones, but little ones. Only because I keep on fighting. You don't see that years out like I am.” (Anne, 62)	
4	D: “Umm, wait and see. I. can't really tell yet, how much I can be at this time. E: Your speech? D: Yes. And, it's not…as fluid as I will like. My understanding always there, but I can never, can get it out. E: The arm is the slowest coming back, by far. D: Yes. But I'm, legs are…in the past two days…E: The last week or so, you've been commenting a lot, just the strength and the feeling in it. D: Yeah, they're stronger” (Daniel, 66 and Emily, spouse)	
5	“I think I did the best I could, with both the arm and leg out of commission, You always think it's gonna be over the next morning or something, so that was kind of disappointing that didn't happen. But the people I worked with were all very positive and supportive, so that's good.” (Cherry, 77)	Acceptance takes time, even with support
6	“Well I do think that having data of what else is going on, you're working with it in therapy, you know, and getting muscles to activate like it would on the other side, being able to learn to identify that little stuff is highly valuable. And I would really like that precision- to be able to be very precise about what is happening.” (Jill, 77)	Identifying small changes with precision is important, hard to tell if something is working
7	“I think if you could see the muscle movement and if you could see that changing over time, that would be really motivating. To know that you're actually doing something and it's working, cause a lot of time, it's hard to tell” (Emily, spouse)	
8	D: “No, but I…use…I don't know. E: I think for me, it depends how long you were seeing nothing. If you worked on it for weeks or months and you still weren't seeing anything, that could be a little [discouraging]. D: Yeah E: But the potential for seeing progress, when there wasn't before, that could be motivating, or worth a try. D: Yes.” (Daniel, 66 & Emily, spouse)	Potential benefits outweigh being discouraged
9	“If [technology] were in the home you get a lot more better because I like these [exercises], I do these every morning. Because you go to another therapist and they give you forty-five minutes and that's it…and then the week next you stay the same thing over and over, you don't get enough time” (Jane, 68).	Role of technology in the home for monitoring recovery and activity
10	“When I got home, they told me, do these exercises, and I did it some, but probably not as much as I should have, and I didn't really understand…And if we would have been monitoring while I was doing them, and they had seen how I wasn't really doing a whole lot, I might have done more, cause I was willing to do more, you know, I just didn't know to do more. And I ended up with a DVT. If [a system] gave you feedback that you were doing something, I think that would have really helped me.” (Jill, 77).	
11	“Always remember that for people like us, time and energy is the thing that beats you down, it really is. Appointments are also kind of logistically challenging. It takes us three hours just to go for a doctor's appointment. Getting ready, down the e-chair into the garage, getting transferred, driving down and then of course they're always late. So, things that you could do at home, remotely…” (Archie, spouse)	The transition home is complicated and tiring, simplicity is key
12	“We have one of those [e-stim units] at home too, that we use on his arm. It's hard finding just the time and when we can both do it, and we're tired a lot (laughs). After a rough day, you know, he gets really tired from the therapy so even though it seems like it wouldn't be that hard to just sit down and do it, it seems to be for us though.” (Emily, spouse)	
13	“Usually when you have a stroke, they figure that you're not competent for your own care. And they not only assume it, they project that out to you and everybody else around you. Which is hard, because you have a tough time communicating, and you can't function, and you're frightened. We became our own advocates, for technology and everything else.” (Ben, 64)	Learning to advocate, using the technology that is available
14	“You have to be able to adapt and provide your own things for yourself. Whether it's training your body or just figuring out how to do it yourself. Whether it's with adaptive technology or with something you use to train yourself. It may be simple technology, but using what's available.” (Anne, 62)	
15	“Another thing you might try would be using the data from this [technology] to improve the system. So you're covered and you can go work with the therapists who do the clinic and have them incorporate that into some of the stuff that they're doing, to show how the muscles are affected, something like that might be a really big training device, and a way to get insurance to understand, because you could see a lot of data” (Ben, 64)	Data showing incremental progress could improve the system
16	“It's a pretty significant investment. Cause of course I'm sure none of this is covered by insurance. If you look at it, think about a Botox injection, I mean, they're incredibly expensive. The insurance company gets billed five thousand dollars every time I have injections…and you know, I would say it helps on the margins, but I don't think it's been miraculous. And I think you run into the same sort of stumbling block with something like [sEMG]. Does it really have the potential to be a game changer? I think a lot of people are looking for what is gonna be a game changer. What's really gonna fix me? At least, that's how I thought about it at first, for better or worse.” (Duke, 49).	

*This table encapsulates relevant example quotes from the results which supported the development of Theme 2: “Tracking Incremental Progress over Days or Years is Important”*.

All participants also highlighted the challenges with transitioning back home after rehabilitation, and a desire to have more connectivity and more technology in a home setting. For example, sEMG could play a role during time at home outside therapy, or as a means to monitor and prevent further medical complications resulting from inactivity (Table 3, Quotes 9, 10). Both caregivers also noted the challenges that arise with the transition home, whether that be fatigue, logistics, or time for technology or other recovery activities (Table 3, Quotes 11, 12). Ultimately, for both treatment decisions as well as technology decisions, participants stressed the importance of involving the stroke survivor at every step, even in the early stages, to self-advocate for their needs (Table 3, Quotes 13, 14).

All participants discussed insurance frustrations during their stroke recovery. Over half the participants (*n* = 6) also noted that data describing these incremental changes noted during recovery could be essential for improving policy and access to neurorehabilitation services as well as technology such as sEMG. However, it was clear to participants that if sEMG will become a justifiable and viable rehabilitation technology, that it must demonstrate its significance to both users and insurance companies (Table 3, Quote 15, 16).

### Theme 3: “Neurorehabilitation Technology Is Cumbersome”

The third theme reflects experiences of stroke survivors with other types of muscle tracking technology, as well as perceptions of current sEMG systems. Participants highlighted issues of cost and funding coverage, time, set-up logistics, and the tension between their desire to optimize time in therapy sessions vs. a willingness to try new technologies that may (or may not) enhance recovery. For example, even for a participant whose previous job was to create and test activity monitors, cost and accuracy were issues (Table 4, Quote 1).

**Table 4:** Theme 3: “Neurorehabilitation technology is cumbersome.”

**#**	**Quote**	**Description**
1	“I wish that I could wear a monitor now, to do a session and see how it is different from what it was, but I haven't wanted to spend the money to get something, and if I was to get one, I don't know that it would work, because I know how much better the monitors that we made were…very medically accurate. (Jill, 77)	Desire for accuracy, lack of money
2	“[Technology] requires a tremendous amount of money and a tremendous amount of effort. It looks cool and shiny and it does a lot of cool things, but how much does it cost? Can a patient afford that? Will insurance cover it because it is so costly? We have the same problems that everyone else does financially. We are looking at income versus outflow.” (Anne, 62).	Out of Pocket cost, re-using supplies, insurance coverage are significant concerns
3	“Now they got my leg on this thing [e-stim/biofeedback], I was a candidate, but Medicare was no good, I had to pay for it all…it was almost six thousand dollars. Then, we had to buy a new computer because it went out and that cost me another four hundred dollars. It's hard, but it's worth it…at least now I can walk. I mean, I have to go very slow, but I can do it. You either gotta work back and then money. Money is a real problem.” (Jane, 68)	
4	“If you have [money], it's fine. If you don't have it…one way we could actually get [our system] to run better would be to use new electrodes every time, but that would get expensive cause you have to keep buying those. So, we re-use them, but you know, the longer you use them, the worse they stick.” (Emily, spouse)	
5	“I read magazines and I say, “Oh, this would be for me.” And we'll go on the computer and see how it does it and everything. I'm always interested in technology and doing it for myself. I have all kind of gadgets that I can do with one hand.” (Jane, 68).	Buy and try mentality
6	“From work, they had this one thing I could try, and I tried it. I didn't really perceive it was helping, so I decided not to keep doing it. And that may have been helpful, but it's hard to know…maybe this is what you get… you don't really know whether it's helpful or not.” (Jill, 77).	
7	“You know, we are always trying to solve problems, there's not always good solutions for us. It's totally just looking for answers to our problems. What's going to help me recover? Whether that's a garbage can or whether it's these little [sEMG] tools. I'm willing to look at it and I'm willing to invest not only my time, my effort, that if I find the right tool, I'm willing to acquire it as an investment.” (Anne, 62)	
8	“I just bought you a Fitbit, I thought that would be motivating with the walking and the steps. And we have a treadmill, so we're not there yet, but that's something we're hoping to use once he's strong enough. D: Well, it's…really beginning stages” (Emily, spouse, and Dan, 66)	Low tech solutions or lay technology use
9	It just doesn't cut it. I hear it's not accurate. It counts your steps, but it won't count half a step. Where if you have a short step like I do, then it doesn't count ‘em. And they tried the heart rate monitor and it doesn't work. I was not impressed.” (Ben, 64)	
10	“You'd put electrodes on your arm and then they actually gave me a laptop that had like a game on it. But it was one game and it was just basically moving one thing around. I mean, it was pretty cool, but it was a hassle to put the electrodes on and taking them off and getting them in the right place. They just sharpied on a mark where they were supposed to go. (Duke, 49).	Hassles with logistics, sensor placement, need for caregiver assist with setup and operation
11	“And the logistics of putting things on or off, That's a show stopper a lot of times. Cause I have to do it, of course. She can't do it if it's on her right arm. There's a lot to do just to get her set up.” (Archie, spouse)	
12	“E: And [e-stim], it's something he can't do by himself. So, I have to be available to do it with him, and then, you know, it's just very touchy so you have to replace [the pads] a lot during the thing, so it's not just like we can slap it on and let it go. We're both kind of there the whole time, so it takes a lot longer than it should, I think. D: What…have a turn on and be working E: And stay on. Exactly (laughing). It's not even the length of time, it's just having to fix it all the time. Makes us both crazy!” (Emily, spouse, and Dan, 66)	
13	“It's hard because at first we would draw around the electrodes, but we get home, and if your arm's in a different position, it's not exact, and then you forget. You take a shower and they're all gone. And I took lots of pictures, you know, I'm there with my pictures trying to figure it out (laughs). This second round though, I feel better, at least about this stuff, than I did the first time. But really, it's a lot of just doing it and then if you can't get it to work, you go in, and they show you again.” (Emily, spouse)	Being creative but needing training refreshers
14	“Sticking anything on and off just gets old after a while, right? So, anything they would come up with that just sits on the skin and can do it, I mean, I think it'll be a real leap forward. I mean, you only have fun ripping the hair out of your arm so many times (laughter) before you sort of just say, ‘Well, what's the point of this!” (Duke, 49)	Hassle of stickers
15	“[They're] not pretty. I mean unless you put little rhinestones on it!” (Anne, 62).	Aesthetics may matter to some
16	“If it gives you a display, where you had something you can train yourself to, so if you're looking at something you can say, “Oh boy! I'm doing good because the pulses are the same on both legs instead of oh, this one was just going like that,” so you get a video or an audio feedback of some kind.” (Archie, spouse)	Needs for the future: multisensory feedback, multiple output styles
17	“If a graph was explained to me, I would love the graphs. I think with [others] you probably need characters or “yay” or something, or a positive sound coming out.” (Cherry, 77)	
18	“If I'm trying, it meets me halfway, with a combination of visual and sensory [feedback]. You know, all the senses, Touch may be another option too” (Anne, 62).	
19	“I'd like something that has a little GPS finder, so there's some cool attraction that individuals may want. Are you more of an outdoor person, is there a flashlight involved? A flashlight would be handy too, you know, Are my shoes under here?!” (Ben, 64)	Multifunctional capacity
20	“Your next level is, is it voice-adaptable? Will it activate by voice? Voice activation would be ideal, because you can connect or sync up to the phone, or ring the doorbell, lock the door, you know, whatever you pick. Maybe color coding - does it turn blue when it's activated, or it turns yellow when you've achieved such a level.” (Anne, 62)	
21	“So, what you really want to do is have it integrated into some sort of central controller so that it could send you a text when you activated the muscle you wanted. Or an email, who knows what the next best thing will be. Tweet you, who knows! Set up a Twitter account for your muscle!” (Duke, 49).	
22	“I think it's about the quality of the technologies that are really out there sort of fully commercialized and fully on the market. And there's a lot of stuff that's kind of in conceptual phases or in beta test or in research studies, but there's not really that much that is sort of fully vetted and fully out there in the market to choose from. If something was demonstrably good at making improvements, I think it would be good if that was available, but I just think there's sort of a dearth of stuff available.” (Duke, 49).	Where the industry is at, and where it can go

*This table encapsulates relevant example quotes from the results which supported the development of Theme 3: “Neurorehabilitation technology is cumbersome”*.

All participants had purchased some type of rehabilitation technology out of pocket, and described similar sentiments about cost, maintenance, and reuse of supplies (Table 4, Quotes 2-4). Overall, the participants described a “buy and try” mentality, resulting in both successes and failures with technology (Table 4, Quotes 5-7). However, despite a willingness to search out and try, most participants (*n* = 7) expressed a preference for lower tech adaptive devices, or more ubiquitously available lay technology. Over half (*n* = 6) of the participants had tried wrist-worn commercial fitness trackers but reported mixed results. Those that responded favorably noted the device's potential for motivation, and those with less favorable opinions cited a lack of accuracy or customizability (Table 4, Quotes 8, 9). When sharing their experiences with sEMG or related technologies such as electrical stimulation, participants described the challenging logistics and cumbersome nature of these rehab technologies as a major barrier to use. Finding the correct sensor placement and need for caregiver assistance for setup were mentioned by half (*n* = 5) the participants (Table 4, Quotes 10-12). Training on proper placement and use of muscle technologies was also reported as a barrier by all the participants who had previously used wearable sensing devices (*n* = 5), even when self-cueing was performed by taking photographs or drawing on the skin. Participants noted the need for refresher training with their therapists when using this technology (Table 4, Quote 13).

Trends emerged, however no consensus from the participants was reached when discussing the benefits and drawbacks perceived from the demonstration of four sEMG systems. In general, participants felt the Delsys Trigno™ was a better research tool and impractical for clinical or home use, which reflects what it and other research-grade systems were designed for. Participants appreciated the flexibility and low profile of the ESS tape sensors and the Biostamp®, but preferred the Myo™ in terms of simplicity and ease of placement without the use of stickers. Half the participants raised the issue of difficulty removing sticker backing and applying sensors one-handed (Table 4, Quote 14). Aesthetics was less of an issue, with only one participant commenting about the sensor appearance (Table 4, Quote 15). Consensus was reached, however, in regard to user-centered feedback. All participants felt that a significant improvement for the future of sEMG systems would be the ability to incorporate real-time, multisensory feedback with flexible methods for receiving signal data outputs that are meaningful to the user (Table 4, Quotes 16-18).

Participants also had some lofty goals for the future of creative sEMG system design and multifunctional capacity. Design ideas included built-in lighting, navigation systems, and greater connectivity between body and technology, envisioned as a social media-style account for muscle tracking (Table 4, Quotes 19-21). Ultimately, participants were excited about the potential for sEMG technology to play a meaningful role in their stroke recoveries, despite the existing barriers of current systems and a lack of cost-effective, adaptable, user-centered systems. As one participant notably summarized, the issue with sEMG in rehabilitation populations is largely about product development stage, user knowledge, availability, and impact in a specialized market (Table 4, Quote 22).

## Discussion

Despite a significant body of research that describes the benefits that sEMG technology may provide in better understanding stroke recovery and enhancing neurorehabilitation outcomes, as well as standards for systematically implementing sEMG, translation to clinical and community settings has been limited (16, 18, 35, 41, 42). One aspect of this complex landscape is patient technology acceptance. Thus, it is important to more closely examine the perspectives of stroke survivors and their families, as central stakeholders in neurorehabilitation, in regard to sEMG technology features and functions. The recovery stories, setbacks and successes, and perceptions and presence of technologies represented in the themes that emerged from this study can play a central role in improving the translational capacity of sEMG systems.

First, our participants noted that little or no muscle monitoring technology was used during the acute and subacute phases of their recovery, these experiences underscore previous work documenting slow uptake of sEMG technologies outside research environments (15, 16). However, stroke survivors and their families indicated that this would be an ideal time to trial sEMG technology, when frustration and fear about return of muscle function is most prevalent and sEMG may detect muscle activity that is not visible or palpable. This early technology intervention is also supported in the literature, and capitalizes on the principles of neuroplasticity routinely cited as drivers of clinical practice in current stroke rehabilitation (43, 44). Further, given that detectable sEMG activity has been seen in flaccid limbs of stroke survivors days after stroke and prior to onset of voluntary muscle contraction, having this resource more readily available in acute recovery phases could serve to build hope and motivation for stroke survivors, in addition to providing information to the medical team about neural pathway integrity (45). Interestingly, participants in this study also discussed the relative “downtime” during recovery, despite their willingness to work on exercises or activities outside of scheduled therapy visits. Rehabilitation clinicians have similarly noted these challenges that come with a relatively passive institutional rehabilitation culture, where stroke survivors have limited opportunities to practice real-world functional skills during down time from therapy or medical cares—which could signal a clinical practice gap in which sEMG may offer novel and individually tailored activity challenges to promote recovery (22).

Second, at home and in the community, stroke survivors demonstrated creativity, and resilience in adapting to their changing abilities, using a combination of high and low technology to improve participation and access. This is consistent with previous literature that describes the processes by which physical resilience is demonstrated following stroke, in part by participating in the hard work of rehabilitation, as well as capitalizing on technologies that may provide access or motivation during recovery (46, 47). However, while technology use with low tech tools such as adapted cutting boards or assistive mobility devices was ubiquitous, perceptions, and use of high-tech tools, including lay technologies for tracking fitness and activity, were quite mixed. These results are also consistent with previous literature describing general interest and excitement combined with skepticism about the features and function of rehabilitation technology (19–21). Further, while most participants were exposed to muscle tracking and training technologies such as electrical stimulation or biofeedback, use was inconsistent and often abandoned due to personal cost, cumbersome set-up, or lack of progress, which is also a common theme in previous work. This presents a unique challenge to clinicians to critically examine how and when these technologies are introduced, as well as to designers and manufacturers of sEMG to understand user and clinician perspectives in early development stages, respond to the relative simplicity and aesthetic appeal of lay technologies, and simultaneously address the precision and adaptive requirements to meet rehabilitation needs.

Third, the participants most strongly highlighted the need for technology to provide both significant and meaningful results. Current sEMG technology had potential in their eyes, and many participants were willing to try, but the financial investment and learning curve were possible barriers, especially if it did not result in impactful information or change from their own point of view. From a perspective of stroke survivor as consumer, this issue is likely one of the most important considerations for future sEMG system design and function. Interestingly, a lack of meaningful outcomes is a frequently cited reason for rehabilitation technology abandonment, however, user-centered, participatory design and implementation strategies are still not widely used in the development of such technologies, especially with older adults (48–51). It is important to consider, however, that sEMG systems may be less like traditional rehabilitation technologies but more closely resemble other wearable biomedical sensors that are engrained in routine clinical care, such as those that monitor heart rhythm (EKG) or brain activity (EEG). These systems also provide valuable results to clinicians and users, but do not require any action by the user aside from passively wearing the sensors. There appears to be a gap in research exploring user acceptance of these similar devices (52), but it remains unclear whether acceptance is not viewed as a major concern, whether differing perspectives may be due to the nuance of sEMG having the potential to elicit action or provide real-time feedback to users, or whether it is simply representative of a unique timeline and trajectory for clinical translation that sEMG may come to enjoy in the future. Regardless, this further highlights the need for additional collaboration between users, clinicians, and engineers during technology development and deployment processes.

Finally, the results of this study point critically to issues of knowledge and understanding of current rehabilitation evidence. Participants discussed the need for further information and education- both for themselves and their families, but also for their healthcare providers- in regard to the benefits and potential outcomes that may be enhanced by sEMG technology. Lack of knowledge or training, time, and self-confidence, as well as a need for meaningful therapeutic outcomes surrounding use of sEMG systems are indeed themes that have been reported previously from the perspective of clinician stakeholders, who often become technology gatekeepers during stroke rehabilitation (28, 35). This is concerning, given the extensive body of literature and standardized guidelines from the SENIAM project that support sEMG as a valuable rehabilitation tool, upon which comprehensive clinician training programs could be built (17, 18). Practical solutions to fill this knowledge gap for clinicians already exist, such as the American Board of Physical Therapy Specialties certification in Clinical Electrophysiology, but could also take the form of international multidisciplinary working groups, further expansion of electrophysiology content in professional rehabilitation training, and vetted teaching and learning modules that extend to a greater number of clinical practice areas.

In addition to technology recommendations from members of their rehabilitation team, a majority of participants largely sought out solutions on their own or at the suggestion of other stroke survivors in their peer groups, which are viewed as an important aspect of recovery (53). Improving education and information sharing among clinicians and stroke survivors appears to be a pathway by which sEMG could achieve greater uptake, provided a clear and compelling message about its utility during recovery could be delivered. This will be a challenge, given the difficulties with system change and novel technology implementation that have been reported in healthcare literature (36, 37). However, there is a unique opportunity to learn from these perspectives and use them to drive product and process improvements. By listening to stakeholders, it is possible for a re-branding of the potential of sEMG technology as a valuable tool that has the capability to provide a rapid, non-invasive, and data-driven look at post-stroke muscle activity which can impact prognostic outcomes, service recommendations, education, and empowerment for stroke survivors and their families.

### Study Limitations

Although the stories shared by stroke survivors and their families who participated in this research provide a much-needed perspective on sEMG technology, there are several limitations to this study. First, the participants represent a small, convenience sample contained within a single metropolitan area, and may not represent the diverse perspectives of a larger or randomized group of stroke survivors. While there was a wide range of ages, genders, stroke types, and rehabilitation courses represented among our participants, all but one individual was Caucasian. Additionally, while a standard set of factual information was shared about four sEMG systems, this is not representative of all available sEMG technologies, so participant perspectives presented here are limited to these systems only. Further, given interview time constraints and to avoid fatiguing the participants, the interviewer did not fully connect or operate the systems in real-time. Participants had the opportunity to physically interact with the sensors, observe signal printouts, and verbally or visually attend to a feature comparison chart. Further work in this area should aim to mitigate these limitations, by intentionally recruiting racially diverse participants, conducting interviews across a wider geographic area, and involving the users in real-time set up and implementation of the sEMG systems over a longer study period to obtain perspectives after full immersion in the processes. Future research should also combine user and clinician perspectives together during rehabilitation care and further assess clinician training in neurorehabilitation technology to determine how closely perspectives align and how therapeutic relationships may affect responses to sEMG technology. Finally, while these perspectives are useful, further user, clinician, and engineering collaboration *before* technology development and deployment will strengthen resulting outcomes, as it is less helpful to constructively critique sEMG systems once they are already commercially deployed.

## Conclusion

Perspectives of individuals with neurologic injuries and their caregivers are one central piece of a broader discussion of factors influencing improved translation of sEMG technology use into clinical settings. The stroke survivors in this study felt that sEMG would be a useful tool for motivation and acquisition of objective data, but that the user interface would have to be simple, available in multiple formats based on the preferences of the user, and provide meaningful feedback for participation in real-world activities, not just exercises. Participants highlighted essential features of sEMG systems, including low cost, flexibility, intuitive and independent use and interpretation, disposability, and comfort. Further translation of sEMG technology for neurorehabilitation into clinical and community environments holds promise, but sEMG system design and user interface needs refinement, and training and education opportunities for clinicians to leverage sEMG technology throughout all phases of rehabilitation following stroke is warranted. Including stroke survivors directly in translational efforts, particularly in creating sEMG system outputs and feedback that is meaningful and motivating to users, is essential to improve uptake in both clinical and community environments.

## Data Availability Statement

The raw data supporting the conclusions of this article will be made available by the authors, without undue reservation.

## Ethics Statement

The studies involving human participants were reviewed and approved by The University of Washington Human Subjects Division. The patients/participants provided their written informed consent to participate in this study.

## Author Contributions

All authors contributed meaningfully to the preparation of this manuscript. HF conducted all the interviews. HF and CP completed primary data analysis. KS and KP completed secondary data analysis. All authors contributed to the writing and editing of the manuscript and equipment used in the study is owned by the Steele Lab in the Department of Mechanical Engineering at the University of Washington. Photos of sEMG systems were taken by HF and are provided courtesy of the Steele Lab. All authors contributed to the article and approved the submitted version.

## Conflict of Interest

The authors declare that the research was conducted in the absence of any commercial or financial relationships that could be construed as a potential conflict of interest.
